# Orthostatic hypotonia as a probably late sequela of SARS-CoV-2 infection in a patient provided with palliative home care: a case report

**DOI:** 10.1186/s40001-022-00685-0

**Published:** 2022-04-29

**Authors:** Agnieszka Kluczna, Elżbieta Mularska, Tomasz Dzierżanowski

**Affiliations:** 1 Laboratory of Palliative Medicine, Department of Social Medicine and Public Health, Medical University of Warsaw, Oczki 3, 02-007 Warsaw, Poland; 2 Observational and Infectious Disease Ward, Clinical Specialist Hospital in Chorzow, Zjednoczenia 10, 41-500 Chorzów, Poland

**Keywords:** Orthostatic hypotension, SARS-CoV-2, COVID-19, Palliative care

## Abstract

**Background:**

The SARS-CoV-2 pandemic has become a challenge for the entire healthcare system. Treatment for COVID-19 includes casual and symptomatic management in the acute phase of the disease and focuses on the treating early complications of the disease. Long-term health consequences of the infection have not yet been fully identified. A special group of patients with comorbidities, including neoplastic disease for whom the interpretation and management of symptoms is a major challenge.

**Case presentation:**

In this case report, we present a 73-year-old woman with recently diagnosed gastric adenocarcinoma in whom we diagnosed orthostatic hypotonia in the aftermath of SARS-CoV-2 infection. We administered thiethylperazine maleate 6.5 mg daily. Additionally, we advised the patient to slowly lift from the recumbent position, raise the headboard, take meals in small portions, and increase fluid intake. These pharmacological and nonpharmacological measures resulted in sustained relief of dizziness and nausea.

**Conclusions:**

The occurrence of orthostatic hypotonia seems a possible late sequela of SARS-CoV-2 infection, and simple measures appeared sufficient to achieve sustained symptom control.

## Background

The SARS-CoV-2 coronavirus infection occurred in December 2019 in Wuhan, China, and rapidly transformed into the COVID-19 pandemic [1]. A total of 151.8 million cases were reported, and over 3.1 million people died worldwide over the first year [2].

The clinical manifestations of SARS-CoV-2 infection are most commonly related to the respiratory system. They usually include cough and dyspnea accompanied by fever. However, an increasing proportion of data indicated that SARS-CoV-2 may also affect other body systems, e.g., the nervous system [3]. Neurological problems were observed in 82.3% of patients hospitalized due to COVID-19 [4]. The most common ones included: headache, myalgia, encephalopathy, anosmia, and dysgeusia or ageusia. Some researchers reported SARS-CoV-2 neurotropism, i.e., the virus's genetic material was found in the brains and brainstems of people and laboratory animals [5, 6].

The exact mechanism responsible for SARS-CoV-2 penetration into the central nervous system has not been fully elucidated yet. However, it was demonstrated that coronavirus might be spread from mechanoreceptors and chemoreceptors located in the lungs and lower respiratory tract into the autonomic center in the brainstem via synaptic connections [7]. The second mechanism involved the blood-borne spread from the systemic circulation into the cerebral circulation, where slower blood flow promoted injury to the capillaries and endothelium exerted by the virus and facilitated access to the brain [6].

Numerous authors reported the influence of SARS-CoV-2, but data concerning the effect of the virus on the autonomic nervous system are scarce [8]. It is due to the fact that the manifestations related to the dysfunctions of the autonomic nervous system (dysautonomia) were usually observed after the acute stage of infection, i.e., during the chronic stage of COVID-19 [9]. The disturbed function of the autonomic system results in orthostatic intolerance syndromes, including orthostatic hypotonia, vasovagal syncope, and postural orthostatic tachycardia syndrome (POTS) [9].

Orthostatic hypotonia and syncope associated with viral diseases may be caused by the loss of gastrointestinal fluid, prolonged bed rest, and the deterioration of cardiovascular and visceral sensory system conditions after acute disease [10].

The paper presents a case report of a woman with gastric adenocarcinoma and the manifestations of orthostatic hypotension in the course of SARS-CoV-2 infection. Medical literature available worldwide includes few case reports of patients with COVID-19 in whom orthostatic hypotonia or other manifestations of dysautonomia occurred. It is because such manifestations usually develop after the acute stage of the infection or after having had the disease when the patients are in an outpatient setting. Therefore, making a correct diagnosis is hardly possible.

## Case presentation

A 73-year-old woman was referred to the gastroenterology clinic because of bleeding from the upper part of the digestive tract, which occurred in December 2019 during an elective hospitalization in the cardiology department, where she underwent one of the stages of coronary angioplasty. The patient record included the following concomitant pathologies: ischemic heart disease, hypertension, mixed hyperlipidemia, atherosclerosis of lower extremity arteries, secondary anemia, status post-coronary angioplasty with the implantation of a DES stent to Cx/OM (31.10.2019) and DES to LAD (03.12.2019). The patient underwent esophagogastroduodenoscopy during hospitalization in the gastroenterology department in February 2020. The examination revealed an irregular polyp covered with necrotic lesions in the distal esophagus, cardia and subcardia regions of the stomach. The test for *Helicobacter pylori* yielded a positive result. The histopathological examination of the specimens confirmed the infiltration with intestinal-type gastric adenocarcinoma, HER-2 negative, CS IV. Abdominal ultrasonography revealed rather massive, hyperechogenic, coalescing, heterogeneous, metastatic-like hepatic lesions and a metastatic lymph node in the area of the left hepatic lobe. The patient was discharged with no clinical symptoms and laboratory signs of recurrent bleeding in the later course of the disease.

Computed tomography of the abdomen performed in March 2020 confirmed multiple hepatic metastases and lymphadenopathy of the retroperitoneal space. The patient was not qualified for surgery. She was qualified for FOLFOX 4 systemic treatment (oxaliplatin, folinic acid, 5-fluorouracil). The patient was admitted to the clinical oncology department after negative SARS-CoV-2 test. The patient received the first and only course of chemotherapy. She was administered iron isomaltoside (1000 mg) and darbepoetin alfa (500 µg) due to anemia (hemoglobin 9.1 g/dL) and low iron concentration in the blood (19.0 μg/dL). Neutropenic fever prophylaxis involved the administration of filgrastim 48 million IU for 5 days. According to the information obtained from the family, the patient had reported malaise and nausea 7 days after discharge, but she was unable to receive medical attention due to the organizational chaos in healthcare-associated with the COVID-19 pandemic. Twelve days after discharge, she was admitted to the infectious disease ward with a fever over 38 °C, nausea, vomiting, and dehydration. The patient underwent the PCR test of the swab collected from the posterior pharyngeal wall. Due to the possibility of developing the complications of systemic treatment in the form of neutropenic fever, empirical antibiotic therapy and growth factors were introduced. After a negative SARS-CoV-2 PCR result was obtained, the patient was transferred to the clinical oncology department to continue treatment. The patient received two units of leukoreduced red blood cell concentrate because of her anemia (hemoglobin 7.9 g/dL). Computed tomography angiography was performed to rule out pulmonary embolism because of multiple risk factors of venous thromboembolism and a high level of d-dimers. On day 21 of hospitalization, the patient’s auto- and allopsychic orientation deteriorated, her blood pressure increased to 200/100 mmHg, and she experienced a tonic seizure. The magnetic resonance of the brain revealed no ischemic foci or metastatic lesions. Antiepileptic treatment was introduced—the patient was administered levetiracetam at a daily dose of 500 mg.

On day 16 of hospitalization in the oncology department, another swab was collected from the patient because of a confirmed coronavirus infection in one of the inpatients. The result was positive for SARS-CoV-2 infection, so our patient was transferred to the infectious disease department. On admission, the patient was not fully oriented. She was bradyphrenic with memory disorders. She had grade 4 performance status on the Eastern Cooperative Oncology Group (ECOG) scale, normal body temperature, and no clinical signs of infection. The physical examination revealed blood pressure of 146/74 mmHg, heart rate 86 bpm, oxygen saturation 90%, and as for abnormal findings—considerable hepatomegaly. Laboratory findings were as follows: C-reactive protein 18 mg/L, hemoglobin 11.3 g/dL, platelets 370 × 10^3^/µL, leukocytes 6.22 × 10^6^/µL, creatinine 0.85 mg/dL, sodium 131.0 mmol/L; potassium 4.77 mmol/L; chlorides 93 mmol/L. The electrocardiogram revealed a normal cardiac axis. On days 7–9 of the hospitalization in the infectious disease department, the patient's blood pressure increased to the maximum of 188/91 mmHg, and on day 17, the patient fell off the bed and cut her left eyebrow. The course of SARS-CoV-2 infection was mild. The patient had no imaging exercise performed, nor she required oxygen supplementation. She received regular diet and self-served. On day 23 of hospitalization, after two negative SARS-CoV-2 results had been obtained, the patient was discharged from the infectious disease department with the recommendation to continue treatment (clopidogrel 75 mg, omeprazole 40 mg, nadroparin calcium 0.6 mL, quetiapine 25 mg, ramipril 10 mg, indapamide SR 1.5 mg and nitrendipine 10 mg daily).

In May 2020, the patient was referred to palliative home care with severe constipation (no bowel movements for 2 weeks) as the main complaint. The abnormal findings on physical examination were: a bedridden patient, ECOG 4, pallor, a 3-cm cut visible on the left eyebrow, a pressure ulcer with dry necrosis on the left heel, hepatomegaly, blood pressure 100/60 mmHg, heart rate 79 bpm, oxygen saturation 93%. The nutritional status, based on physical examination, was correct. The patient received four meals and at least 1500 mL of fluids per day. Any attempt at raising the headboard over the angle of 45° or sitting up resulted in nausea and dizziness. A syncopal episode was provoked when the patient was ambulated. The manifestations persisted despite the discontinuation of hypotensive drugs and normal fluid intake. On day 15 after discharge from the hospital, the patient was diagnosed with orthostatic hypotension.

The Schellong test was performed to confirm the diagnosis [11]. However, after 30 s of ambulation, the patient reported nausea and dizziness, so the test was discontinued. The Head Upright Tilt Table test was not performed in home setting [12]. No other tests were performed to confirm the dysfunction of the autonomic nervous system because of the lack of consent. The patient was followed up. She was administered thiethylperazine maleate 6.5 mg once daily. At the same time, the nonpharmacological treatment involved a slow postural shift from a recumbent to sitting position, the avoidance of increased abdominal muscle pressure, raising the headboard by 10°–20°, consuming small portions of food, increasing fluid intake [13].

The therapeutic management was verified during a subsequent visit. The intensity of nausea and dizziness was reduced, and the resultant intensity of those symptoms was acceptable. In the patient’s opinion, the adherence to the nonpharmacological instructions contributed to the improvement in her well-being.

## Discussion

Orthostatic hypotonia is defined as systolic pressure reduction by at least 20 mmHg or diastolic pressure reduction by at least 10 mmHg during 3 min after shifting from a recumbent to a sitting position [14]. It is a symptom and not a disease [15]. A blood pressure decrease results from the inability of the autonomic nervous system to reach the appropriate venous return and the constriction of blood vessels sufficient for the maintenance of blood pressure. Such a regulation depends on baroreceptor reflexes, normal blood volume, and mechanisms responsible for excessive blood pooling in the venous bed [16]. The phenomenon usually occurs during sudden ambulation and may be more intense in the morning, which is associated with increased blood pressure in a recumbent position and nocturnal natriuresis. Moreover, non-specific problems are usually present, such as generalized weakness, chronic fatigue, nausea, and headaches. Visual disorders are due to the ischemia of the retina and cerebral occipital lobes. Furthermore, anginal pain is observed. It results from functional myocardial ischemia in patients with normal coronary vessels. Dizziness associated with concentration disorders is a common problem. They deteriorate the quality of life and the ability to perform everyday activities [17]. The ischemia of the trapezius muscle and cervical muscles may trigger pain in the suboccipital, nuchal, cervical, and back areas [17].

The hypoperfusion of the pulmonary apices caused by the dysregulation of ventilation leads to orthostatic dyspnea in patients with orthostatic hypotonia [18]. The loss of consciousness and transient sightlessness occur in severe cases, and in the most severe cases, transient ischemic attacks are observed [19]. The above manifestations are associated with emotional discomfort and anxiety. Two forms of orthostatic hypotonia are distinguished depending on the time passed after ambulation: early orthostatic hypotonia with the onset of symptoms within 3 min after changing the position, and the delayed with the clinical manifestations observed between 5 and 40 min after ambulation [20, 21]. The maintaining of appropriate values of blood pressure during ambulation and in a standing position depends on blood volume in the vascular bed, the coordinated function of vasculo-neural impulses, and humoral response. Ambulation results in the shift of approximately 500–1000 mL of blood to the lower extremities and visceral circulation. Venous return to the heart is decreased, and ventricular filling is diminished, which result in reduced cardiac output and blood pressure. Such hemodynamic changes trigger a compensatory reflex response to restore normotension. The diminished response of the baroreceptors of the carotid sinus and aortic arch leads to the decreased afferent activity of the vagus nerve and the glossopharyngeal nerve. Physiologically, it activates the sympathetic nervous system leading to an increased vasopressin production. As a consequence of those reflexes, the peripheral resistance of venous return and cardiac output is increased, which limits blood pressure decrease [22]. Visceral veins also play an essential role, as they redistribute blood to the systemic circulation as a result of baroreceptor reflexes [23]. If the response is unsuccessful, the patient experiences orthostatic hypotonia and cerebral hypoperfusion, which lead to syncope.

The risk factors of developing orthostatic hypotonia, being a manifestation of dysautonomia, include primary and secondary pathologies of the central and peripheral nervous system, endocrine, metabolic disorders, or electrolyte disorders, hypovolemia, hypochromic anemia, taking hypotensive and psychotropic medications, sympathectomy and hyperbradykininism [17].

According to recently published research, orthostatic hypotonia and other syndromes of orthostatic intolerance were also observed in approximately 3% of the SARS-CoV-2 patients [24, 25]. Orthostatic intolerance syndromes associated with SARS-CoV-2 infection usually occurred during the chronic stage of COVID-19, so they were rather unrelated to autonomic system damage caused by the virus. However, they might be due to an autoimmune reaction. Moreover, autonomic dysfunctions such as orthostatic hypotonia and postural orthostatic tachycardia syndrome were linked to autoantibodies, e.g., towards a-/b-adrenoreceptors and muscarinic receptors [26, 27]. She had a history of gastric adenocarcinoma treated with chemotherapy, chronic anemia, and epilepsy diagnosed immediately before COVID-19 (no discernible lesions within the central nervous system). The patient also had signs of dementia. The diagnosis of SARS-CoV-2 infection was made based on the positive result of the nasopharyngeal swab tested via the PCR method. Despite numerous concomitant diseases, the course of COVID-19 was not associated with respiratory infection manifestations during the hospitalization in the infectious disease department. The exacerbation of encephalopathy was the only problem observed in the patient.

Although the negative Schellong test result did not confirm orthostatic hypotonia, the whole clinical picture supported the diagnosis. The described orthostatic disorders might also be due to prolonged stay in a recumbent position, insufficient fluid intake, the use of hypotensive drugs, hypochromic anemia, or as a paraneoplastic syndrome [28, 29]. However, the onset of those symptoms after having had COVID-19 suggests the possible viral etiology of the syndrome. Some authors reported similar cases of orthostatic intolerance syndromes, including orthostatic hypotonia, associated with SARS-CoV-2 infection [8]. Cohort studies indicated the infectious etiology of POTS and the relationship with autoimmune diseases or disorders [25, 30]. Seemingly, similar mechanisms may occur in the case of COVID-19.

## Conclusions

The asymptomatic initial course of SARS-CoV-2 infection does not rule out the possibility of developing undesirable manifestations associated with the infection after eliminating the virus, such as orthostatic hypotonia. Therefore, it is essential to follow-up convalescents for long-term complications, and the diagnosis of orthostatic hypotonia related to a recent SARS-CoV-2 infection.

## Data Availability

Not applicable.

## Abbreviations

*POTS*: Postural orthostatic tachycardia syndrome
*ECOG*: Eastern Cooperative Oncology Group
