# Correction: Orthostatic hypotonia as a probably late sequela of SARS‑CoV‑2 infection in a patient provided with palliative home care: a case report

**DOI:** 10.1186/s40001-022-00712-0

**Published:** 2022-05-31

**Authors:** Agnieszka Kluczna, Elżbieta Mularska, Tomasz Dzierżanowski

**Affiliations:** 1 Laboratory of Palliative Medicine, Department of Social Medicine and Public Health, Medical University of Warsaw, Oczki 3, 02‑007 Warsaw, Poland; 2 Observational and Infectious Disease Ward, Clinical Specialist Hospital in Chorzow, Zjednoczenia 10, 41‑500 Chorzów, Poland

## Correction: Eur J Med Res (2022) 27:60 https://doi.org/10.1186/s40001-022-00685-0

Unfortunately, in the original publication, the affiliation details of the first author were incorrectly published. The corrected affiliation details of the first author were given in this correction article. The original article [1] has been corrected.
